# Sociodemographic and Socioeconomic Determinants for the Usage of Digital Patient Portals in Hospitals: Systematic Review and Meta-Analysis on the Digital Divide

**DOI:** 10.2196/68091

**Published:** 2025-06-03

**Authors:** Nina Goldberg, Christin Herrmann, Paola Di Gion, Volker Hautsch, Klara Hefter, Georg Langebartels, Holger Pfaff, Lena Ansmann, Ute Karbach, Florian Wurster

**Affiliations:** 1 Chair of Health Services Research in Rehabilitation Faculty of Human Sciences & Faculty of Medicine, Institute of Medical Sociology, Health Services Research and Rehabilitation Science University of Cologne Cologne Germany; 2 Department of Digital Clinical Systems Clinical Affairs and Crisis Management Unit University Hospital Cologne Cologne Germany; 3 Clinical Ethics Committee University Hospital Cologne Cologne Germany; 4 Center for Health Services Research (ZVFK) Faculty of Medicine and Cologne University Hospital University of Cologne Cologne Germany; 5 Institute of Medical Sociology, Health Services Research and Rehabilitation Science Faculty of Medicine and University Hospital Cologne University of Cologne Cologne Germany; 6 Chair of Medical Sociology Faculty of Human Sciences & Faculty of Medicine and University Hospital Cologne, Institute of Medical Sociology, Health Services Research and Rehabilitation Science University of Cologne Cologne Germany

**Keywords:** patient portal, digital divide, hospital, meta-analysis, socioeconomic, sociodemographic, e-Health

## Abstract

**Background:**

Digital patient portals (PPs) are platforms that enhance patient engagement and promote active involvement in health care by providing remote access to personal health data. Although many hospitals are legally required to offer these portals, adoption varies widely among patients, often influenced by sociodemographic and socioeconomic determinants. Evidence suggests that higher income, education, employment status, and specific age groups correlate with increased portal usage, highlighting a digital divide. This study aims to analyze sociodemographic and socioeconomic determinants affecting digital PP usage, addressing inconsistencies in existing research and contributing to strategies for reducing digital health disparities.

**Objective:**

This study aimed to conduct a meta-analysis of the sociodemographic and socioeconomic factors contributing to the digital divide in the usage of digital PPs.

**Methods:**

A systematic review with meta-analysis was conducted using PRISMA (Preferred Reporting Items for Systematic Reviews and Meta-Analyses) guidelines in PubMed, Web of Science Core Collection, and EBSCOhost. Screening involved 3 reviewers with consensus meetings to resolve discrepancies. Data on sociodemographic and socioeconomic factors and statistical outcomes were extracted, and study quality was assessed using the Mixed Methods Appraisal tool. Results were visualized using forest and funnel plots to assess heterogeneity and publication bias.

**Results:**

A total of 2225 studies were identified through a systematic review, and after title and abstract screening, 17 studies were included in the quantitative and qualitative analysis. The qualitative analysis revealed that younger patients (younger than 65 y) were significantly more likely to use the digital PP, while the meta-analysis revealed that women had a 16% higher likelihood of using the digital PP than men. The relationship between income and digital PP usage was inconsistent, due to different scaling in different studies. A higher level of education was significantly associated with a 37% greater likelihood of using the digital PP in the meta-analysis. In addition, employed patients were 23% more likely to use the digital PP, while married patients had a 13% higher likelihood of using it than unmarried patients. Marital status and employment can be considered as measurable factors of social relationships.

**Conclusions:**

The review confirms that sociodemographic and socioeconomic factors significantly influence the usage of digital PP in hospital care. Marital status shows that social support plays a vital role, with married patients 13% more likely to engage with digital PPs. It is worth noting that social support through connections to society via work or work colleagues can also play an important role as like as a partner at home, with employed individuals being 22% more likely to use digital PPs. Overall, sociodemographic factors, like marital status, primarily affect usage patterns, while socioeconomic factors, like employment, enable access, emphasizing the need for comprehensive support systems to bridge the digital divide in health care.

**Trial Registration:**

German register of clinical trials DRKS00033125; https://drks.de/search/de/trial/DRKS00033125 and PROSPERO CRD42024567203; https://www.crd.york.ac.uk/PROSPERO/view/CRD42024567203

## Introduction

Digital patient portals (PP) are platforms that offer remote access to personal health data and are meant to empower patients to take a more active role in their own medical care [1]. As an example, the ability to make appointments and communicate directly with medical staff via the digital PP can strengthen the sense of personal responsibility in relation to one’s own health [2,3]. Overall, evidence shows that the use of such digital PP does enhance patient engagement, education, and overall care [4,5]. While many hospitals are legally required to offer digital PP through national regulations, patients can choose whether to make use of it. There is a significant difference between patients in the general adoption of digital applications in health care [6,7], which, among other factors, may contribute to increasing social inequalities in health care and suboptimal health outcomes [8]. The usage of digital PP seems to depend particularly on patients’ sociodemographic and socioeconomic status [9]. The most common determinants analyzed in this regard are income, education, and employment for socioeconomic [10], age, gender, and marital status for the sociodemographic determinants [11]. The empirical results show that certain privileged groups of people, with regard to the sociodemographic and socioeconomic status, are more likely to use a digital PP and therefore benefit disproportionately from the benefits described [12,13].

**Figure 1:**
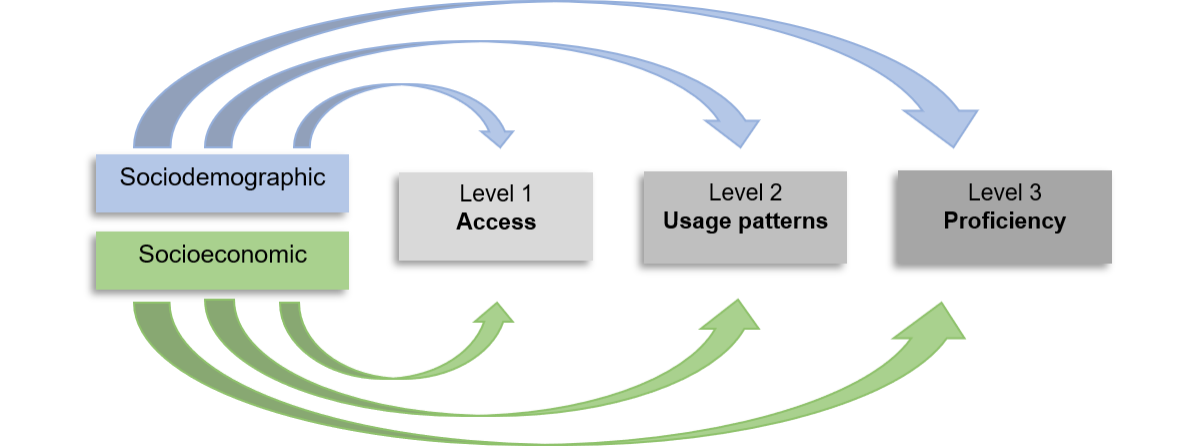
Own illustration according to Deursen and Helsper [19].

This phenomenon is described with the term “digital divide.” People with certain perceptions, capabilities, and characteristics are less engaged with the digital transformation and, as a result, are less able to benefit from the emergence of new digital forms of care [14]. For example, recent studies show that patient age may play a role in the use of digital PP and that digital PP interfaces should be designed to be accessible to all age groups, as older people require more support [15,16]. The digital divide has already been identified in literature and can be observed, for example, in patients with lower levels of education or income and as described above in older patients [17,18]. Deursen and Helsper [19] present a 3-level model of the digital divide in health care, which serves as a basis for combating digital inequality. The first level describes the gap in terms of lack of access to digital tools and resources for internet access, the second level deals with usage patterns, and the third level with the ability to use digital technologies effectively and efficiently to achieve better results on general outcomes. In addition, each of the 3 levels and therefore the digital divide is influenced by socioeconomic and sociodemographic factors [19-21] (Figure 1).

It can be assumed that the digital divide also applies to the adoption of digital PP, since research has identified certain socioeconomic and sociodemographic determinants of the use of digital PP, as described above [9]. In order to develop strategies to overcome the digital divide and inequalities regarding the usage of digital PP [22], it is of considerable relevance to explore the characteristics of users and nonusers [23] in order to gain insights into which characteristics are related to the use or nonuse of digital PP. However, the evidence is inconsistent regarding the usage behavior of specific sociodemographic and socioeconomic groups. Some studies indicate that younger patients may be more likely to use a digital PP [24,25], while another suggests older patients to be in favor [26]. In this context, a more detailed differentiation would be interesting. Furthermore, some studies suggest that individuals with a lower level of education [27] are more likely to use a digital PP, while other studies indicate that individuals with a higher level of education might be in favor [28,29]. The aim of this study is to provide an overview and an analysis of the sociodemographic and socioeconomic factors that characterize users and nonusers of digital PP. Previous studies have already examined usage behavior regarding sociodemographic and socioeconomic factors, but only under the specific light of other dimensions such as digital health literacy [30] or certain diseases [31] with regard to usage behavior. However, no study or review has focused exclusively on sociodemographic and socioeconomic factors and on the strength of associations with the use or nonuse of digital PP in hospitals and thus on the digital divide. Due to the inconsistent evidence between the individual studies described above and the disagreement as to how strong the influence of individual sociodemographic and socioeconomic determinants is, a meta-analysis is necessary.

## Methods

### Overview

A systematic review with meta-analysis was conducted to answer the research question and is reported based on the latest version of the PRISMA (Preferred Reporting Items for Systematic Reviews and Meta-Analyses) guidelines described by Page et al [32]. Refer to Multimedia Appendix 1 for a detailed list of where to find which items. The study is embedded in the overarching research project “Multiperspective analysis of patient portal use over time - based on the example of digital anamnesis in a best-practice hospital,” which investigates the adoption of a digital PP under the light of the diffusion of innovations theory [33]. The research project is listed in the German register of clinical trials (DRKS00033125) and in the international clinical trials registry platform of the World Health Organization. This review was registered in the PROSPERO database (CRD42024567203).

### Search Strategy and Selection Criteria

The search was carried out in March 2024. Following a sensitive search strategy to identify all eligible studies, several databases were searched, including PubMed (including PubMed, PubMed Central, and MEDLINE), Web of Science Core Collection, and EBSCOhost. The components for the database search were “patient portal” and “socioeconomic factors” or “sociodemographic factors.” A discussion of scientific and practical relevance within the research team (NG, FW, and CH), resulted in 19 related synonyms for the component “patient portal” and another 21 related synonyms for the component “socioeconomic” and “sociodemographic.” A filter was applied to all databases to limit the results to English and German languages and up to the year 2010 in order to exclude far-outdated evidence on this topical issue. The full search term, including synonyms, Boolean operators, number of results, and filters, can be found in Multimedia Appendix 2. Furthermore, 4 meetings were held between the researchers to develop a common understanding of the inclusion and exclusion criteria and to eliminate discrepancies. Disagreements were discussed after each step of the literature review, and agreement was reached on the included studies at the end. The results were screened in 3 steps by 3 researchers (NG, FW, and CH) who included or excluded studies according to the criteria in Textbox 1. In the first step, all titles were screened independently of each other by NG and FW or CH. The abstracts were then screened independently by NG and FW or CH, after which the remaining full texts were assessed by NG and FW or CH. If not, enough information was available or given for a decision, the studies were included in the next step. The screening process was conducted with Rayyan software (Qatar Computing Research Institute) to support collaboration, and included publications were stored in a Citavi library.

**Inclusion and exclusion criteria.**

**Inclusion criteria**

- Patient portal
- In the hospital setting
- Patients >18 years
- Comparison of characteristics of users and nonusers
- Peer reviewed publications with quantitative data analysis
- Used by patients

**Exclusion criteria**

- No patient portal
- Outside the hospital setting
- Patients <18 years
- No comparison of characteristics of users and nonusers
- Publications of secondary literature like reviews, comments, essays, and publications with qualitative data analysis only
- Used by medical staff only

### Data Extraction

Of the included studies, information on the authors, year of publication, country, setting, number of users and nonusers, the sociodemographic and socioeconomic characteristics, with focus on age, gender, marital status [11], and income, employment, and education [10], and the results were extracted. For all studies, statistical results such as CIs, *P* values, or other relevant effect measurements (eg, odds ratio and SD) were extracted if the authors provided them. The results were summarized qualitatively and quantitatively. To decide which studies were eligible, the characteristics and extracted data of each study were tabulated and compared in Microsoft Excel.

### Assessment of the Risk of Bias of the Study

The Mixed Methods Appraisal Tool (MMAT; version 2018) proposed by Hong et al [34] was used to assess the quality of the included studies. The MMAT is a specially designed tool that can be used to assess the quality of different types of studies in the same review, including qualitative, quantitative, and mixed methods studies. All included studies are quantitative nonrandomized. The following questions are used for the MMAT: (1) Is the sampling strategy relevant to address the research question? (2) Is the sample representative of the target population? (3) Are the measurements appropriate? (4) Is the risk of nonresponse bias low? (5) Is the statistical analysis appropriate to answer the research question? Following the recommendations for reporting the results of the MMAT (2018 version), the studies were rated on a scale of 0-5 stars. Each of the 5 conditions assessed was given 1 star if fulfilled, an unclear or unmet condition was given 0. Low-quality studies were not excluded from this review, but the quality of the included studies was presented, and a possible risk of bias based on the MMAT rating was discussed with the research team. The scoring was carried out independently by NG and FW or CH, and any discrepancies were discussed in the research team (NG, FW, and CH).

### Data Analysis

For the systematic review part, a qualitative synthesis was compiled for the content and results of all included studies. For the additional meta-analysis, all statistical analyses were performed with the software RStudio (version 4.2.2; Posit) and the packages *metafor* [35] and *meta*. The risk ratio (RR) is used to interpret the results. Regression coefficients were estimated with 95% CIs using a random effects model [36] and a common effects model to check the sensitivity of the analyses [37]. The significance level was set at *P*<.05. Heterogeneity due to differences between studies was assessed using the *I*² statistic [38], and a significant heterogeneity was present if the *I*² was >50% [39]. A sensitivity analysis was conducted to assess the robustness of the meta-analysis results. Accuracy was reported with 95% CIs. Forest plots were used to visually represent the presence of heterogeneity. Publication bias was assessed using funnel plots [40] (Multimedia Appendix 3).

## Results

### Overview

**Figure 2:**
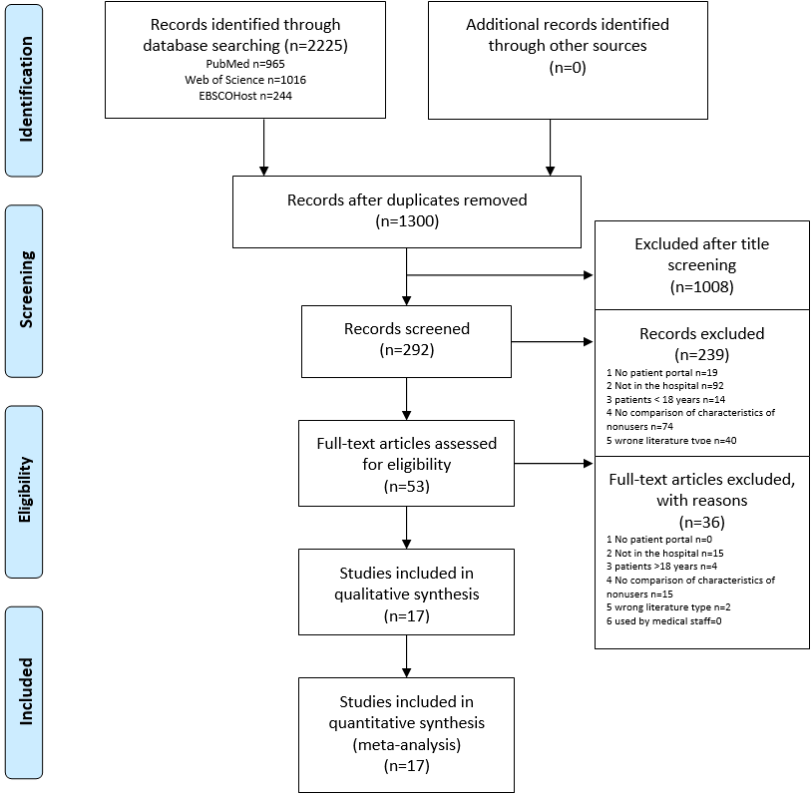
PRISMA (Preferred Reporting Items for Systematic Reviews and Meta-Analyses) flowchart.

The study selection process and the reasons for excluding studies are illustrated as a flowchart in Figure 2. The database searches revealed a total of 2225 studies (PubMed: n=965; Web of Science: n=1016; and EBSCOhost: n=244) with 1300 studies remaining after removing duplicates. After the title screening, the number of included studies was 292, and 53 remained after checking the abstracts for eligibility. The final 17 studies were included in this systematic review and in the quantitative analysis.

All included studies investigated sociodemographic and socioeconomic determinants. Although the hospital setting was an inclusion criterion, the hospital setting varied regarding differences in specialty (eg, transplant station [29], oncology [22], and orthopedics [41]). The number of patients analyzed varied from a minimum of 235 [42] to a maximum of 424,840 [26]. In 2 studies, 2 separate patient groups were analyzed, and different results were found for the individual determinants. These results from Holte et al [41] and Wedd et al [29] were considered separately in the following review and meta-analysis and treated as individual studies in the analysis. Refer to Table S1 in Multimedia Appendix 4 for study characteristics such as users and nonusers of digital PP and the reported sociodemographic and socioeconomic factors. The most frequently analyzed characteristics were age [22-29,41-49], gender [22-29,41-49], and education [23,24,26-29,41,42,44]. Furthermore, 8 studies included the income [23,26,27,41-44,49], 6 studies included employment [22,24,25,29,46,48], and 5 studies examined marital status [23,25,27,29,48]. The included studies were mainly conducted in North America [22-29,41-49]. Furthermore, 1 study was located in Argentina [45], 1 in the United Kingdom [28], and 1 in the Netherlands [24]. No publication bias was detected among the included studies, as indicated by the funnel plots (Multimedia Appendix 3), except for the meta-analysis with regard to gender. Due to asymmetric distribution, which could indicate a publication bias, an Egger test [40] was calculated. However, this test is not statistically significant, and so there is no indication of publication bias.

A meta-analysis was carried out for the factors that were reported comparably in the studies. No meta-analysis could be calculated for the determinants age and income, as the included studies reported their results in different categories or units of measurement. For the other determinants (gender, education, employment, and marital status), not all included studies could be included in the meta-analysis, as not all studies described all factors. For gender, 15 studies [22-26,28,29,41-48], for marital status, 5 studies [23,25,27,29,48], for employment status 6 studies [22,24,25,29,46,48], and for education, 7 studies [23,24,26,28,29,41,44] could be included in the meta-analysis.

Based on the MMAT tool, 9 studies achieved 3 stars [22,25,27,28,41,43-45,47], 3 studies achieved 4 stars [24,46,49], and 5 studies achieved 5 stars [23,26,29,42,48] as an assessment for their quality.

### Age

In 9/17 studies, users were younger according to multivariate statistical models [22-25,27,41,45,47,48]. In 1 out of 17 studies, users were older [26], and 7 out of 17 studies found no statistically significant results [28,29,42-44,46,49]. It was not possible to conduct a meta-analysis due to the inconsistent reporting of patient age in the various studies. The studies used different categories and units of measurement, which led to considerable variability and prevented a direct quantitative consolidation of the data. In a bivariate analysis and multivariate analysis by Balthazar et al [27], users were significantly more likely to be younger. Also, Holte et al [41], Hoogenbosch et al [24], Martinez et al [45], Plate et al [25], Owolo et al [48], and Ochoa et al [47] came to the same conclusion, that is, users were statistically and significantly younger than nonusers. Nielsen et al [46] had the same results in the univariate analysis (users tend to be younger (*P*≤.001), but these were not statistically significant after logistic regression. In the study by Emani et al [23], 85.9% (317/369) of users compared with 46.2% (129/279) of nonusers being younger than 65 years. Patients younger than 65 years also had higher odds of using the digital PP described by Griffin et al [22]. In contrast to the other results, the patients enrolled in the digital PP were older in the study by McFarland et al [26]. Wedd et al [29], Tome et al [42], Neves et al [28], Lockwood et al [44], Glosser et al [43], and Ukoha et al [49] found no statistically significant results.

### Gender

**Figure 3:**
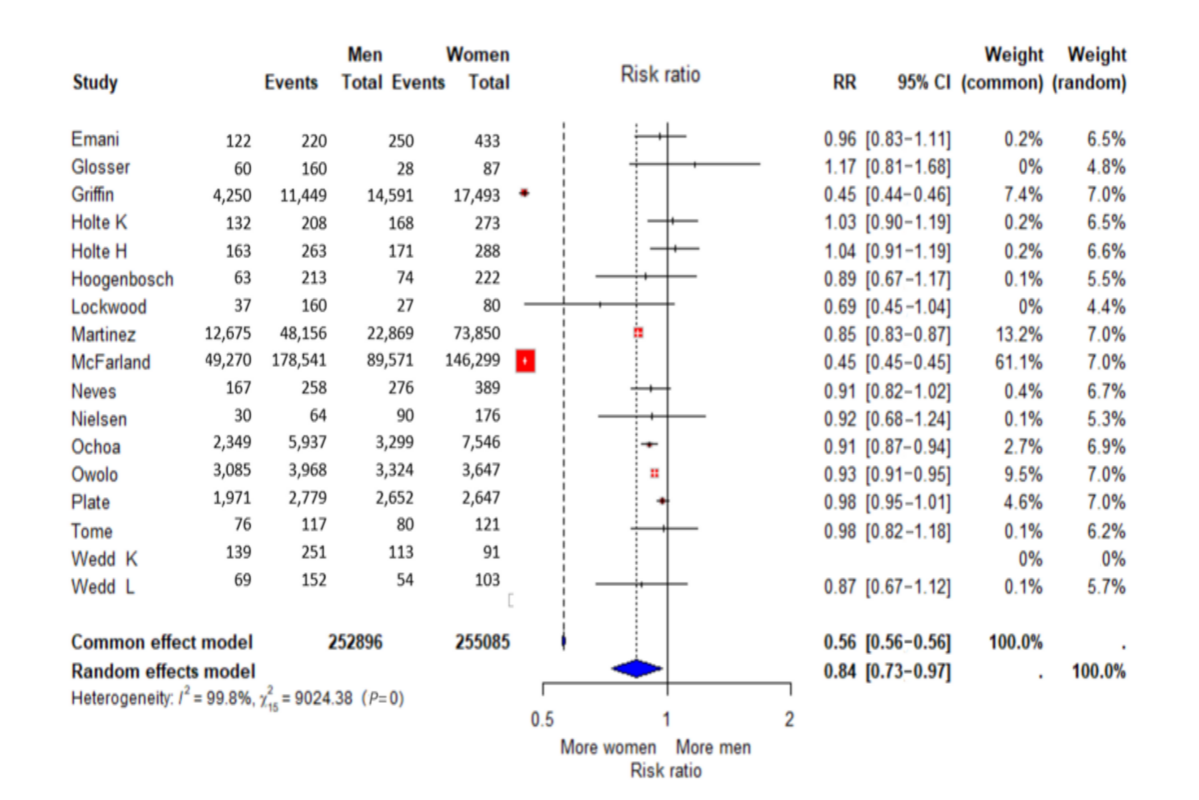
Forest Plot “Gender” ([22-26,28,29,41-48]).

In 5 out of 15 studies, users were more likely to be female than male according to multivariate statistical models [22,26,45,47,48]. Out of 15 studies, 10 found no statistically significant results. Griffin et al [22] reported that men had lower odds than women for using the digital PP. The univariate analysis by McFarland et al [26] showed patients enrolled in the digital PP were more likely to be female, and Martinez et al [45] also confirmed this with a significant association to digital PP use. Ochoa et al [47] and Owolo et al [48] also found that women use the digital PP more frequently than men. Emani et al [23], Glosser et al [43], Holte et al [41], Hoogenbosch et al [24], Lockwood et al [44], Neves et al [28], Nielsen et al [46], Plate et al [25], Tome et al [42], and Wedd et al [29] found no statistically significant results in relation to gender and usage (Figure 3).

A total of 252,896 of the patients were male and 255,092 were female. The male patients formed the experimental group, and the female patients formed the control group in the meta-analysis. The RR random effects model was 0.84 (95% CI 0.73-0.97) and was statistically significant (*P*<.001). This means that male patients have a 16% lower probability of using the digital PP than female patients (RR 0.84). According to *I*², the variance explanation between the studies was 99.8%. Since an *I*² of 99.8% indicates a high heterogeneity of the studies and the funnel plot shows a strong asymmetry (Multimedia Appendix 3), an Egger test was calculated in RStudio. As this did not provide a significant result (*P*=.08), no sufficient statistical evidence of publication bias can be provided, although slight bias cannot be ruled out [40].

### Income

In 5 out of 8 studies, users had a higher income than nonusers according to multivariate statistical models [23,26,41-43]. Out of 8 studies, 3 found no statistically significant results [27,44,49]. It was not possible to conduct a meta-analysis due to the inconsistent reporting of income data in the various studies. The studies used different categories and income thresholds to represent income, which led to considerable variability and prevented a direct quantitative consolidation of the data. In the analysis by Balthazar et al [27], users were more likely to live in neighborhoods with lower average household incomes. Ukoha et al [49] and Tome et al [42] came to the opposite conclusion, that is, patients with a low income were significantly less likely to enroll in the digital PP. These findings by Balthazar et al [27] and Ukoha et al [49] were not statistically significant in the multivariate analysis. Holte et al [41] and McFarland et al [26] found that users of the digital PP had a higher income than nonusers. In total, 56% (182/325) of users reported by Emani et al [23] had a total household income of US $75,000 or more compared with 33% (75/227) of nonuser with a total household income of US $75,000. Glosser et al [43] were also able to show that patients with an income level greater than US $40,000 were more likely to use the digital PP than those with an income less than US $40,000. Lockwood et al [44] found no statistically significant result in relation to income and using the digital PP.

### Education

**Figure 4:**
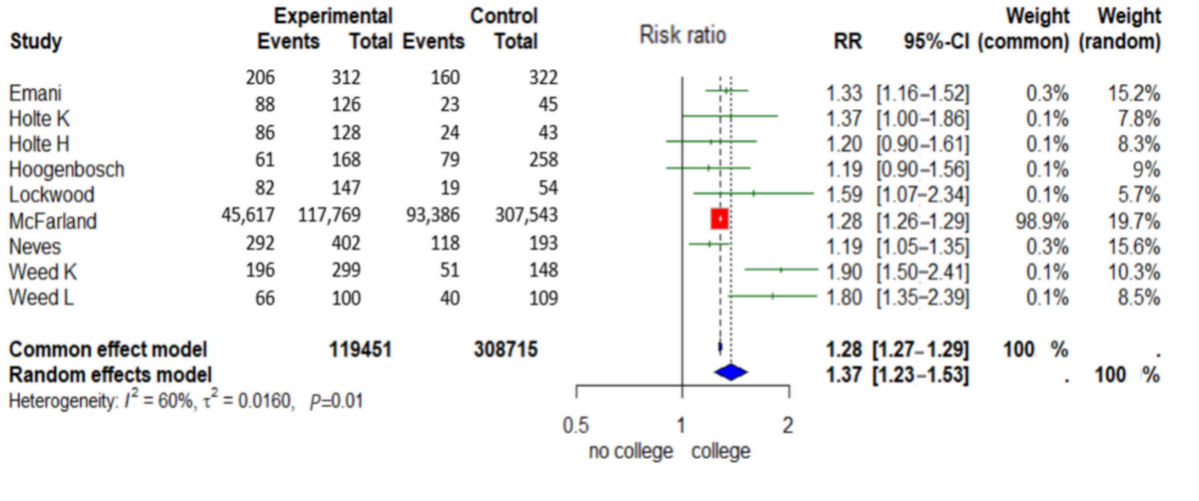
Forest plot “Education” ([23,24,26-29,41,42,44]).

In 5 out of 9 studies, more users had an advanced education than nonusers according to multivariate statistical models [26,28,29,42,44]. Out of the 9 users, 1 had no advanced education than nonusers [27]. Of the 9 studies, 3 found no statistically significant results [23,24,41]. The level of education had a statistically significant influence on the use of the digital PP in 6 out of 9 studies [26,28,29,42,44]. Except for Balthazar et al [27], who found that users of the digital PP had a statistically significant lower education level than nonusers, all concluded that users had a higher level of education [23,26-29,42,44]. Holte et al [41], Hoogenbosch et al [24], and Emani et al [23] found no statistically significant results on the association between education and digital PP use according to multivariate statistical models (Figure 4).

In order to standardize the educational levels and make them easier to interpret, the *International Standard Classification of Education* (*ISCED*) was used. According to the *ISCED-11* definition, an advanced education level is defined as a short-cycle tertiary education or higher [50].

A total of 119,451 patients had an advanced level of education, and 308,715 patients below an advanced level. The patients with an advanced education level formed the experimental group, and those with an education level below advanced formed the control group. The RR random effects model was 1.37 (95% CI 1.23-1.53) and was statistically significant (*P*=.01). This means that patients with an advanced education level are 1.37 (37%) more likely to use the digital PP than patients below the advanced education level. According to *I*², the variance explanation between the studies was 60%. Furthermore, 2 studies were excluded from the quantitative analysis because they did not report on the presence or absence of a college degree [27,42].

### Employment

**Figure 5:**
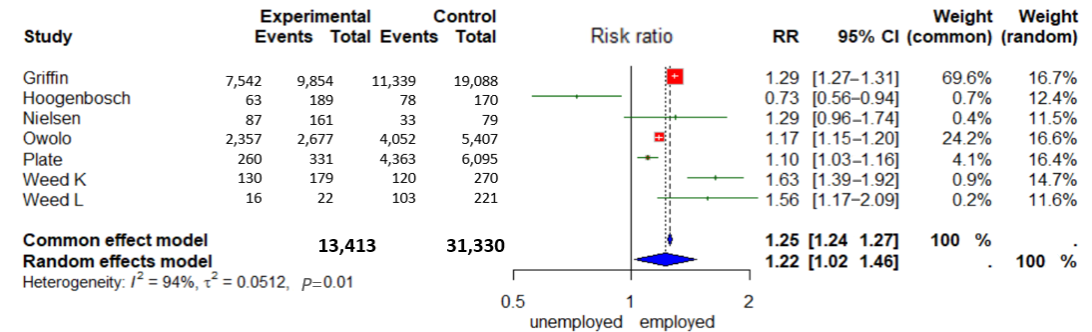
Forest plot “Employment” ([22,23,25,29,44,48]).

In 4 out of 6 studies, more users were employed than nonusers according to logistic regression or multivariate analyses [22,25,29,48]. In 1 out of 6 studies, the users were less often retired [23], and 1 out of 6 studies found no statistically significant result [44]. In their study, Wedd et al [29] collected data from 2 different groups of patients, which are presented in this paper as 2 studies. So, two-thirds of the studies came to the same conclusion that patients who were not employed or retired were statistically significantly less likely to use a digital PP. The majority of users in each study were employed full-time (Figure 5).

A total of 13,412 employed and 31,330 unemployed patients were included. The employed patients formed the experimental group and the unemployed the control group. The RR random effects model was 1.22 (95% CI 1.02-1.46) and was statistically significant (*P*<.01). This means that employed patients are 1.22 (22%) more likely than unemployed patients to use the digital PP. According to *I*², the e-variance explanation between the studies was 94%.

### Marital Status

**Figure 6:**
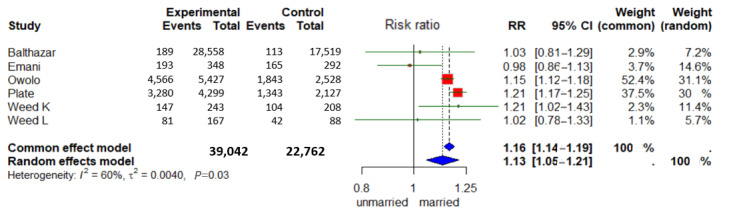
Forest plot “Marital Status” ([23,25,27,29,48]).

In 2 out of 5 studies, more users were likely to be married or in a domestic partnership than nonusers according to logistic regression or multivariate analyses [25,48]. Out of 5 studies, 3 found no statistically significant results [23,27,29]. Wedd et al [29] collected data from 2 different groups of patients, which are presented in this paper as 2 studies (Figure 6).

A total of 39,042 married and 22,762 unmarried patients were included in the meta-analysis. Married persons formed the experimental group, and unmarried persons formed the control group. The RR random effects model was 1.13 (95% CI 1.05-1.21) and was statistically significant (*P*=.03). This means that married patients are 1.13 (13%) more likely to use the digital PP than unmarried patients. According to *I*², the variance explanation between the studies was 60%.

## Discussion

### Principal Findings

This systematic review and meta-analysis confirmed the influence of sociodemographic and socioeconomic factors on the usage of PP, a central aspect of digitalization in hospital care. The study focused on gender, age, and marital status as indicators for sociodemographics and income, and education and employment as proxies for socioeconomics.

### Sociodemographics

With regard to gender, no significant results were found in 10 of the 15 studies. This suggests that the gender-specific effect may be less pronounced than previously assumed in the literature. Although the results of the meta-analysis show that men use the PP 16% less frequently and therefore may have a greater need for support, this should not be overinterpreted as the sole factor in view of the many nonsignificant results of the included studies. The finding that younger patients are more likely to use digital PP than older patients (9/17 studies) is not unexpected. This digital divide in terms of usage can be explained by a number of factors, including the fact that younger patients grow up with technical innovations and may therefore use them intuitively in a health care context. Of greater interest is the finding that 7 out of 17 studies could not find any significant correlations between usage and age. One possible explanation is that older patients are now also more tech-wise, as more technical knowledge is required in many areas of life. It is also conceivable that many older people receive social support from their environment. In the course of the digital divide, age is still a factor, but probably no longer as decisive as assumed. Nevertheless, it could be interesting for future research to differentiate the age groups into even smaller groups in order to find more specific usage patterns of individual generations. Age and gender, therefore, do not appear to be the only factors influencing the use of digital PPs. In terms of marital status, the meta-analysis also showed that married patients were 13% more likely to use the digital PP than unmarried patients. It can therefore be concluded that marital status is an influential determinant of the use of digital PP among the sociodemographic factors analyzed.

If living with a partner is associated with a higher probability of use, social support could be a relevant factor for the use of digital PP, because marital status is often used in studies as a proxy for social support [51]. One explanation could be that it is rather the amount of social support that each patient has, in the form of a partner, at their disposal, that can close the digital divide. This means that the presence of social support in the home environment could also have an impact on the use of digital PP. At home, people can discuss problems, receive support in using digital PP, and be reminded and encouraged to use them. In this case, the social support of a partner could be referred to as emotional, informational, and instrumental social support [52].

### Socioeconomics

Of the 9 studies that analyzed education level, 5 showed an association between higher education (advanced education level) and use of the digital PP. In addition, the results of the meta-analysis also suggest that the likelihood of using the digital PP is 37% higher if the patient has an advanced education level or higher. Closely related to this, the employment status of patients may be relevant in explaining their use. The meta-analysis showed that the employed group was significantly more likely to use the digital PP (22%). The results on income were highly inconsistent. Based on the evidence that low socioeconomic status is associated with poor use of health care resources [53], it is expected that the use of digital PP will also be suboptimal in low socioeconomic patient groups. It can be argued that access to the internet or digital devices is also influenced by the economic situation of the patient. Level 1 of the “Third-Level Digital Divide Model” addresses this situation and thus reiterates the considerable importance of establishing support measures for financially disadvantaged patients [19,20]. It is therefore worth noting that while the socioeconomic determinants are of great importance, social support through connections to society via work or work colleagues can also play an important role, similar to the sociodemographic determinants. For example, work colleagues can help out with missing digital devices, or work devices can be used. Similar to a partner at home, work colleagues can therefore provide all forms of social support [52], and they can also be trusted persons in health matters through regular contact, as well as being a support person when it comes to using a digital PP. Based on the results of the meta-analysis, it is assumed that not all determinants affect each level, but that the socioeconomic factors could affect level 1 of the model and enable access to digital PP. On the other hand, usage patterns as level 2, may be more likely to be influenced by social support, which could come from a partner at home or from work colleagues. It should be emphasized that each determinant could influence each level of the model. The fact that the sociodemographic and socioeconomic factors analyzed here do not address level 3 “proficiency” of the model is due to the focus on general use by patients and not on effective and efficient proficiency use to achieve better outcomes. It remains to be clarified what role personal competencies, influencing factors such as health literacy or self-management, play and whether these can be considered and integrated in further studies. The existing literature already suggests that health literacy could act as a possible mediator between socioeconomic status and the likelihood of using digital services in the hospital [20]. Socioeconomic determinants such as education or age as a sociodemographic determinant could also influence the proficiency of patients, for example, accessing and understanding for example digital clinical notes.

**Figure 7:**
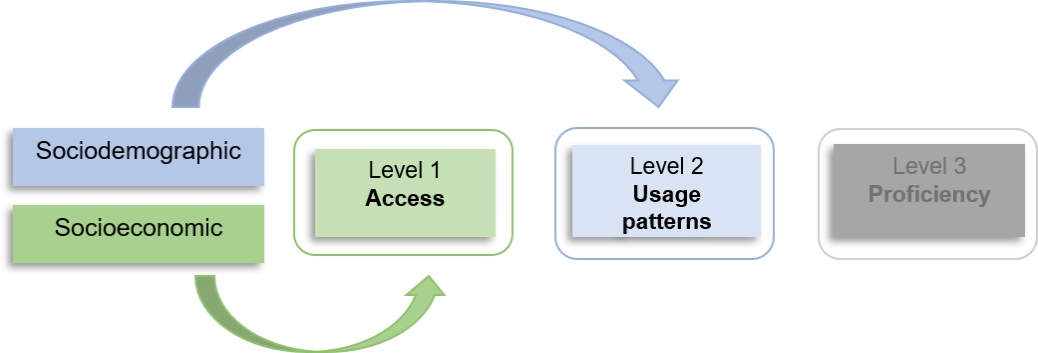
Own illustration according to van Deursen and Helsper [19].

The results of the meta-analysis suggest the following pathways (Figure 7). Further research should test these pathways and incorporate the other pathways, because these were not tested in this paper, in order to confirm the model. Based on the results of the meta-analysis, it could be of great interest to investigate the causal relationships between employment, education, marital status, and the resulting social support and personal skills such as health literacy. This should also be considered in future research. It can also be concluded that if social support from a partner or a job can be responsible for a use, but a patient does not have this, a solution must be found within the health care system itself, because the literature already describes social support as a catalyst for accessing and using digital information and communication [54]. In this case, more support is needed from hospital staff, such as social workers, to compensate for the lack of social support from family, partner, or work colleagues [55]. They can also provide instrumental support by offering practical assistance on-site in setting up and using the digital PPs or by helping with technical problems. Informational support for patients could be provided by social workers in the form of education about the benefits of digital PPs, but also in the form of easy-to-understand instructions or workshops for patients and their families. This could also help to improve patients’ health literacy. This social support could also address level 3 “proficiency” of the model by Deursen and Helsper [19] and achieve a more effective and efficient use of digital PPs in hospitals. Increased staffing for support needs to be a policy issue, otherwise, there is a risk that patients with little private support will be lost in the digital divide.

It is striking that all the countries included in this study are pursuing a national digital health strategy. Either through financial incentives, regulations, or policies. In the Netherlands, every hospital offers a digital PP, and while there is only a percentage estimate of the prevalence of digital PPs in the United States, it can be assumed from the regularization that digital PPs are also very widespread in Argentina and the United Kingdom (Table 1). This would also explain why only studies from these countries were included. It would be interesting to carry out a direct international comparison in further research and also to examine countries that do not have national policies.

**Table 1:** Country comparison.

Country	Legal background	Implementation rate
United States	There is no legal requirement for hospitals to offer a patient portal, but regulatory pressure and financial incentives [56].	90% (2018) [57].
Netherlands	Digital health is seen as part of the regular health care system [58].	Every hospital in the Netherlands offers a digital patient portal [59].
United Kingdom	NHS^a^ Care [60].	No exact survey for implementation but “digital first” option [61].
Argentina	Digital health strategy [45].	No exact survey for implementation.

^a^NHS: National Health Service.

### Limitations

It was not possible to conduct a meta-analysis on income and age as determinants, for which a pooled result would have been interesting due to the inconsistent reporting of study results. In addition, further social determinants such as social support or digital competence have not been analyzed in this review, which could have explained further correlations between usage and socioeconomic and sociodemographic factors. This should be taken up in further studies, and research should provide explanations and see which other determinants have a significant influence on usage behavior. Another limitation is the small number of socioeconomic and sociodemographic determinants. The determinants used were selected according to their frequency for measuring socioeconomic and sociodemographic status. For further research, it would be useful to also include the geographical area of the hospitals and the patients’ places of residence in the search terms, as these are likely to have an impact on both the availability of digital PP and the social status of the patients. In addition, it should be emphasized that in this study, marital status was interpreted as a prerequisite for the presence of a partner or family support. This does not correspond to the modern living arrangements of many patients and should be further evaluated in future research. For example, the presence of a partner without being married or the intention to have children. Other family members can also play a supportive role. Another limitation is that the type of employment is only distinguished between full-time and not full-time employment. In the future, it would be important to further differentiate the various “not employed” categories, as this could include retirement, part-time work, or actual unemployment. In general, only studies in German and English were included. In this matter, it is questionable how many studies in other languages might exist, since the majority (14/17) of the studies were based in North America. Only 2 European studies were included, which indicates a significant lack of research on studies outside North America on the use of digital PP. This raises the question of why this is the case and whether there is an insufficient amount of research on digital PP, or whether there has been a lower implementation of digital PP in Europe in general. This could be due to differences in data protection. It would be advisable to investigate this in a separate study and work out the differences. It is a matter of fact that in 2019, nearly all hospitals enabled a digital PP in America [62]. It should also be noted that the heterogeneity between the studies analyzed in a meta-analysis is very high, and publication bias is possible. This can be attributed to the fact that there are major differences in the study conditions, characteristics, and implementation. As different functions of PP and different patient groups are analyzed, the high heterogeneity is not surprising. Furthermore, despite statistical tests for bias in the meta-analysis due to the heterogeneity of the included studies, this cannot be ruled out. In the future, further meta-analyses should be calculated for the same functions with the same patient population.

### Conclusion

The meta-analyses conducted have shown that higher socioeconomic status and specific sociodemographic determinants increase the likelihood of using the digital PP. Contrary to what was assumed, the importance of gender and age was lower regarding the use of digital PP, while marital status, employment, and level of education are stronger predictors than assumed. The application of the model by Deursen and Helsper [19] highlights that socioeconomic status has an impact on level 1 of the model, which describes general access to digital tools and interventions. However, social support from partners (marital status) or work colleagues (employment) appears to promote usage patterns (level 2) of digital PP, as assumed in earlier studies [63]. If social support from partners or work colleagues is not available as a resource, hospital employees could fill the social support gap and provide informal, emotional, and instrumental social support to patients. The key could be to increase the number of employees such as hospital social workers. Nevertheless, future studies and policies should also consider other factors, such as social support, and thus build further bridges between the sociodemographic and socioeconomic determinants and factors such as support through social support. This additional bridge could address the proficiency (level 3 of the model), which concerns the effective and competent use of digital innovations. The state of research in Europe on the use of digital PP is lacking behind and needs to be expanded, with the aim of enabling patients to use a digital PP in hospital, with special emphasis on sociodemographic or socioeconomic determinants, in order to ensure adequate care in hospital and to further close the digital divide.

## Appendix

Multimedia Appendix 1 Search strategy.

Multimedia Appendix 2 Funnel plots.

Multimedia Appendix 3 PRISMA (Preferred Reporting Items for Systematic Reviews and Meta-Analyses) checklist.

Multimedia Appendix 4 Characteristics of the included studies.

## Abbreviations

ISCED: International Standard Classification of Education
MMAT: Mixed Methods Appraisal Tool
PP: patient portal
PRISMA: Preferred Reporting Items for Systematic Reviews and Meta-Analyses
RR: risk ratio
