# Supplementary material for: Sociodemographic and Socioeconomic Determinants for the Usage of Digital Patient Portals in Hospitals: Systematic Review and Meta-Analysis on the Digital Divide
Source: J Med Internet Res. 2025 Jun 3;27:e68091. doi: 10.2196/68091 (PMC12174889; doi:10.2196/68091)
Supplement: Multimedia Appendix 1 [file jmir_v27i1e68091_app1.docx]

| patient portal | AND | socioeconomic |
| --- | --- | --- |
| patient web portal |  | social status |
| patient internet portal |  | sociodemographic |
| digital health service |  | socially disadvantaged |
| ehr portal |  | social determinants |
| electronic health record portal |  | health equity |
| electronic portal |  | digital divide |
| electronic health portal |  | social disparities |
| health portal |  | Disadvantaged |
| digital health interventions |  | Disparities |
| web portal |  | Social inequalities |
| Personal health record |  | Social equity |
| Patient Health record |  | social |
| Online Record Access |  | SES |
| Open Notes |  | Socioeconomic status |
| OpenNotes |  | Inequity |
| Shared Medical Record |  | Inequality |
| Accessible electronic health record |  | Disparity |
| Patient-Accessible Electronic Health Record |  | Soci* |
|  |  | Social class |
|  |  | Socio-economic |
|  |  | demographic |

Limits: English / German , since year 2010

**1a. PubMed 28.02. 965 studies**

(("patient portal"[Title/Abstract]) OR ("patient web portal"[Title/Abstract]) OR ("patient internet portal"[Title/Abstract]) OR („digital health service" [Title/Abstract]) OR ("ehr portal"[Title/Abstract]) OR ("electronic health record portal"[Title/Abstract]) OR ("'electronic portal"[Title/Abstract]) OR ("electronic health portal"[Title/Abstract]) OR ("health portal"[Title/Abstract]) OR („digital health interventions"[Title/Abstract]) OR ("web portal"[Title/Abstract]) OR („personal health record“[Title/Abstract]) OR („patient health record“[Title/Abstract]) OR („online record access“[Title/Abstract]) OR („open Notes“[Title/Abstract]) OR („OpenNotes“[Title/Abstract]) OR („Shared medical record“[Title/Abstract]) OR („Accessible electronic health record“[Title/Abstract]) OR („patient-accessible electronic Health Record“[Title/Abstract])) AND ((socioeconomic*[Title/Abstract]) OR ("social status"[Title/Abstract]) OR ("sociodemographic"[Title/Abstract]) OR („socially disadvantaged*"[Title/Abstract]) OR („social determinants"[Title/Abstract]) OR („health equity"[Title/Abstract]) OR („digital divide"[Title/Abstract]) OR („disadvantaged“[Title/Abstract]) OR („disparities“[Title/Abstract]) OR („social disparities“[Title/Abstract]) OR („social inequalities“[Title/Abstract]) OR („social equity“[Title/Abstract]) OR („social“[Title/Abstract]) OR („SES“[Title/Abstract]) OR („socioeconomic status“[Title/Abstract]) OR („inequity“[Title/Abstract]) OR („inequality“[Title/Abstract]) OR („Disparity“[Title/Abstract]) OR (Soci*[Title/Abstract]) OR („social class“[Title/Abstract]) OR („socio-economic“[Title/Abstract]) OR („demographic“[Title/Abstract]))

**1b. Web of Science 28.02. 1.016 studies**

#27 AND #29

((((((((((((((((((((((TS=("socioeconomic")) OR TS=("social status")) OR TS=("sociodemographic")) OR TS=("socially disadvantaged")) OR TS=("social determinants")) OR TS=("health equity")) OR TS=("digital divide")) OR TS=("social disparities")) OR TS=("disadvantaged")) OR TS=("disparties")) OR TS=("social inequalities")) OR TS=("social equity")) OR TS=("social")) OR TS=("SES")) OR TS=("socioeconomic status")) OR TS=("inequity")) OR TS=("inequality")) OR TS=("disparity")) OR TS=(soci*)) OR TS=("social class")) OR TS=("socio-economic")) OR TS=("demographic"))

AND

((((((((((((((((((TS=("patient portal")) OR TS=("patient web portal")) OR TS=("Patient Internet Portal")) OR TS=("digital health service")) OR TS=("EHR portal")) OR TS=("electronic health record portal")) OR TS=("electronic portal")) OR TS=("Electronic Health Portal")) OR TS=("Health Portal")) OR TS=("digital health interventions")) OR TS=("Web Portal") OR TS=("personal health record")) OR TS=("patient health record")) OR TS=("online record access")) OR TS=("open Notes")) OR TS=("OpenNotes")) OR TS=("Shared medical record")) OR TS=("accessible electronic health record")) OR TS=("patient-accessible electronic health record"))

**1c. Ebsco Host 244 studies-> imported 91 because ebsohost automatically removes duplicates**

("patient portal“ OR "patient web portal“ OR "patient internet portal“ OR „digital health service" OR "ehr portal“ OR "electronic health record portal" OR „electronic portal" OR "electronic health portal" OR "health portal" OR „digital health interventions" OR "web portal" OR „personal health record“ OR „patient health record“ OR „online record access“ OR „open Notes“ OR „OpenNotes“ OR „Shared medical record“ OR „Accessible electronic health record“ OR „patient-accessible electronic Health Record“) AND (socioeconomic* OR "social status" OR "sociodemographic" OR „socially disadvantaged“ OR „social determinants" OR „health equity" OR „digital divide" OR „disadvantaged“ OR „disparities“ OR „social disparities“ OR „social inequalities“ OR „social equity“ OR „social“ OR „SES“ OR „socioeconomic status“ OR „inequity“ OR „inequality“ OR „Disparity“ OR Soci* OR „social class“ OR „socio-economic“ OR „demographic“)

https://web.p.ebscohost.com/ehost/resultsadvanced?vid=9&sid=1f54f542-5fcb-4129-9503-6f4cd115d0c2%40redis

https://search.ebscohost.com/login.aspx?direct=true&db=asn&db=h9h&db=h9i&db=h9j&db=h9k&db=h9m&db=pdh&db=psyh&db=bas&db=bth&db=nlebk&db=eoh&db=eoah&db=eric&db=f3h&db=geh&db=guh&db=8gh&db=lxh&db=msn&db=cmedm&db=kah&db=mzh&db=nsm&db=ddu&db=phl&db=pdx&db=bwh&db=ram&db=rip&db=snh&db=sih&db=dfn&db=dfg&db=tnh&db=cin20&bquery=TI+(%26quot%3bpatient+portal%e2%80%9c+OR+%26quot%3bpatient+web+portal%e2%80%9c+OR+%26quot%3bpatient+internet+portal%e2%80%9c+OR+%e2%80%9edigital+health+service%26quot%3b+OR+%26quot%3behr+portal%e2%80%9c+OR+%26quot%3belectronic+health+record+portal%26quot%3b+OR+%e2%80%9eelectronic+portal%26quot%3b+OR+%26quot%3belectronic+health+portal%26quot%3b+OR+%26quot%3bhealth+portal%26quot%3b+OR+%e2%80%9edigital+health+interventions%26quot%3b+OR+%26quot%3bweb+portal%26quot%3b+OR+%e2%80%9epersonal+health+record%e2%80%9c+OR+%e2%80%9epatient+health+record%e2%80%9c+OR+%e2%80%9eonline+record+access%e2%80%9c+OR+%e2%80%9eopen+Notes%e2%80%9c+OR+%e2%80%9eOpenNotes%e2%80%9c+OR+%e2%80%9eShared+medical+record%e2%80%9c+OR+%e2%80%9eAccessible+electronic+health+record%e2%80%9c+OR+%e2%80%9epatient-accessible+electronic+Health+Record%e2%80%9c)+AND+(socioeconomic*+OR+%26quot%3bsocial+status%26quot%3b+OR+%26quot%3bsociodemographic%26quot%3b+OR+%e2%80%9esocially+disadvantaged%e2%80%9c+OR+%e2%80%9esocial+determinants%26quot%3b+OR+%e2%80%9ehealth+equity%26quot%3b+OR+%e2%80%9edigital+divide%26quot%3b+OR+%e2%80%9edisadvantaged%e2%80%9c+OR+%e2%80%9edisparities%e2%80%9c+OR+%e2%80%9esocial+disparities%e2%80%9c+OR+%e2%80%9esocial+inequalities%e2%80%9c+OR+%e2%80%9esocial+equity%e2%80%9c+OR+%e2%80%9esocial%e2%80%9c+OR+%e2%80%9eSES%e2%80%9c+OR+%e2%80%9esocioeconomic+status%e2%80%9c+OR+%e2%80%9einequity%e2%80%9c+OR+%e2%80%9einequality%e2%80%9c+OR+%e2%80%9eDisparity%e2%80%9c+OR+Soci*+OR+%e2%80%9esocial+class%e2%80%9c+OR+%e2%80%9esocio-economic%e2%80%9c+OR+%e2%80%9edemographic%e2%80%9c)&lang=de&type=1&searchMode=And&site=ehost-live
