# Supplementary material for: Sociodemographic and Socioeconomic Determinants for the Usage of Digital Patient Portals in Hospitals: Systematic Review and Meta-Analysis on the Digital Divide
Source: J Med Internet Res. 2025 Jun 3;27:e68091. doi: 10.2196/68091 (PMC12174889; doi:10.2196/68091)

**Funnel Education**


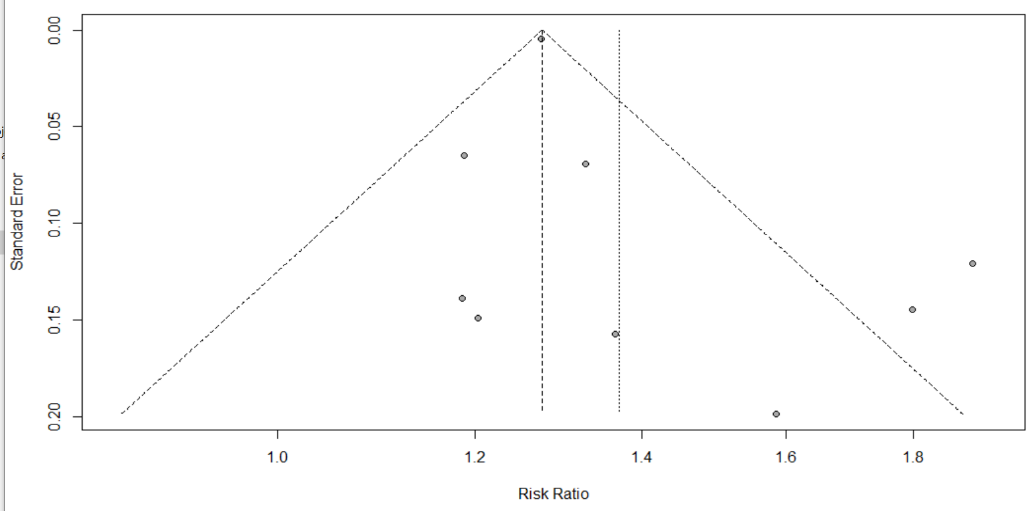


**Funnel Employed**


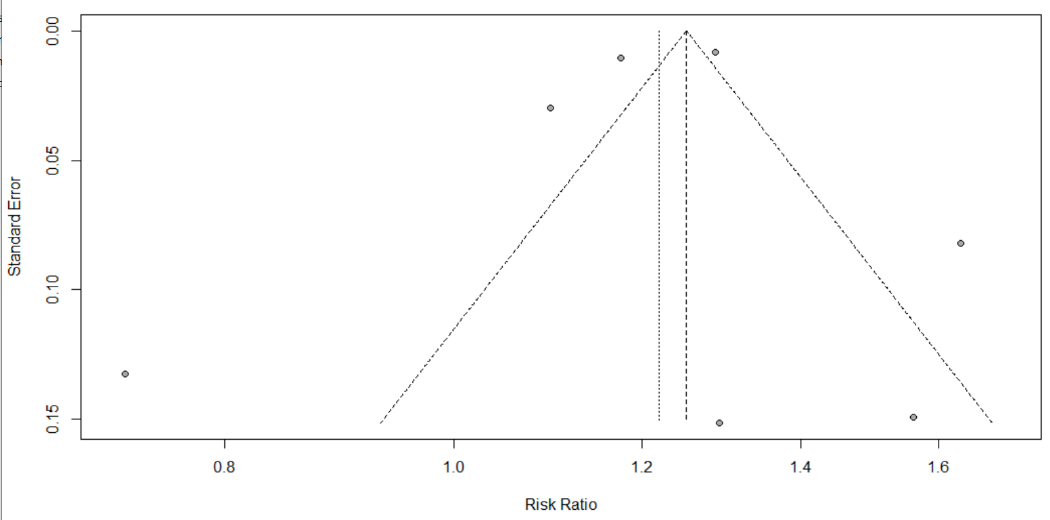


**Funnel Gender**


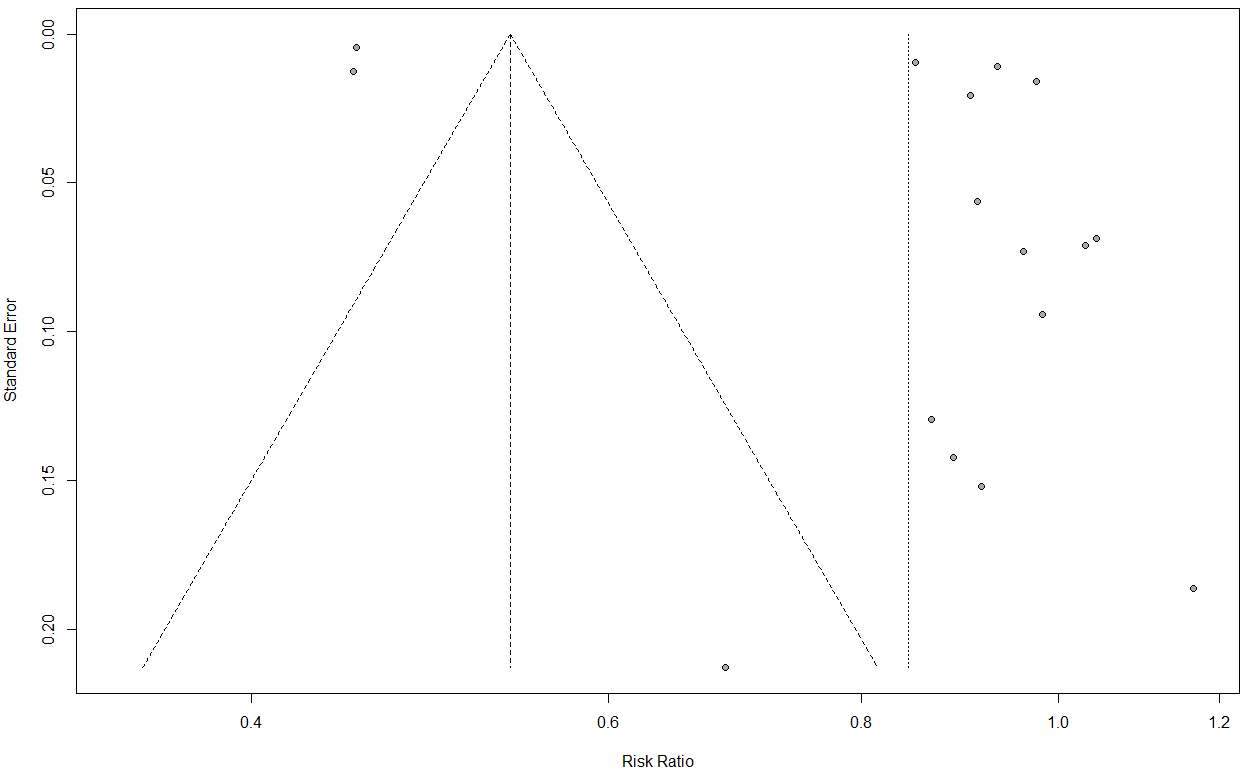


Egger Test:

Test result: t = 1.91, df = 14, p-value = 0.0762

Bias estimate: 13.7246 (SE = 7.1679)

**Funnel Married**


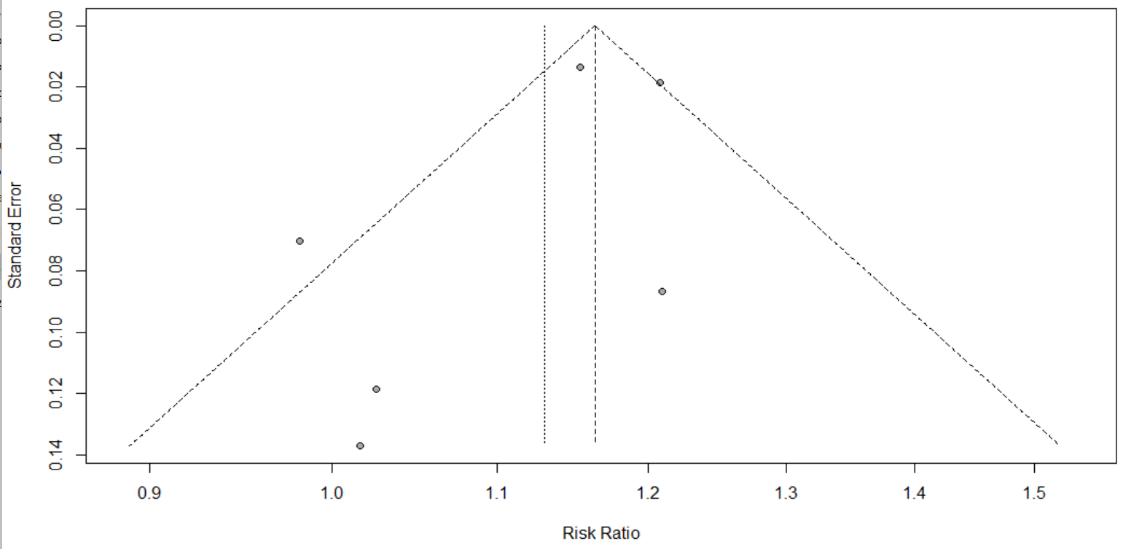

Supplement: Multimedia Appendix 2 [file jmir_v27i1e68091_app2.docx]
