# Supplementary material for: Sociodemographic and Socioeconomic Determinants for the Usage of Digital Patient Portals in Hospitals: Systematic Review and Meta-Analysis on the Digital Divide
Source: J Med Internet Res. 2025 Jun 3;27:e68091. doi: 10.2196/68091 (PMC12174889; doi:10.2196/68091)
Supplement: Multimedia Appendix 4 [file jmir_v27i1e68091_app4.docx]

**Table S1.** Characteristics of the included studies.

| Authors | Country | | Setting | Users, n | Non-users, n | Age | Gender | Income | Education | Employment | Marital status | When as user defined? | MMAT^a^ |
| --- | --- | --- | --- | --- | --- | --- | --- | --- | --- | --- | --- | --- | --- |
| Balthazar et al [27] | United States | | Female patients undergoing screening | 302 | 45,781 | Younger users: 53.4 vs 60.3 y, *P*<.001  Younger users: OR^b^ 0.94, 95% CI 0.93-0.96, *P*<.001 | NA^c^ | Lower average incomes (US $46,782 vs US $48,366 *P*=.03)  Median household income was NSly. | Lower education 35.9% vs 37.7% at or above college *P*=.02)  Lower education OR 0.98, 95% CI 0.97-1.00, *P*=.02) | NA | NS^d^ | Use of a PP^e^ to self-schedule screening mammography | * * * |
| Emani et al [23] | United States | | No specific setting | 372 | 281 | 85.9% (317/369) of users were younger than 65 y compared to 46.2% (129/279) of nonusers (*P*<.001) younger  OR 3.75, 95% CI 2.17-6.46, *P*<.001 | NS | 56% (182/325) of users reported a total household income of US $75,000 or more compared with 33% (75/227) of nonuser (*P*<.001) higher income  OR 1.87, 95% CI 1.17-3.00, *P*<.001 | 56.3% (206/366) of users had a 4-year college degree or more compared to 39.6% (106/268) of nonusers (*P*<.001)  Not statically significant in the multivariate model | NA | NS | General adoption or use of the PP | * * * * * |
| Glosser et al [43] | United States | | Kidney transplant adult recipients | 88 | 159 | NS | NS | Those earning greater than US $40,000 were more likely to use the portal than those with an income less than US $40,000 (OR 2.95, 95% CI 1.36-6.42, *P*=.006)  Higher income  OR 2.51, 95% CI 1.08-5.83, *P*=.033) | NA | NA | NA | Used the portal at least once | * * * |
| Griffin et al [22] | United States | | Adult patients who received cancer treatment | 18,881 | 10,061 | Younger than 40 y (AOR^f^ 2.56, 95% CI 2.21-2.98, *P*<.001) between 40 and 65 y (AOR 1.81, 95% CI 1.68-1.97, *P*<.001) had higher odds of using the portal  Middle age was associated with higher odds of portal use than older age (AOR 1.27, 95% CI 1.13-1.43, *P*<.001) | Men had lower odds than women for using the PP  AOR 0.85; 95% CI 0.80-0.89 *P*<.001 | NA | NA | Patients not working for pay (AOR 0.41, 95% CI 0.38-0.45, *P*<.001) and those who had retired (AOR 0.64, 95% CI 0.59-0.70, *P*<.001) had lower odds of using the portal | NA | Used the portal at least once | * * * |
| **Holte et al [41]** | United States | | Patients who underwent primary THA^g^ or TKA^h^ | 640 | 399 |  |  |  | NS | NA | NA | Opted-in PP | * * * |
|  | Knee |  |  |  |  | User: younger (95% CI 0.09-0.27, *P*≤.001) | NS | Users had a higher income than those who do not use (*P*≤.001) |  |  |  |  |  |
|  | Hip |  |  |  |  | Users: younger (95% CI –0.07 to –0.01, *P*=.016) | NS | Users had a higher income than those who not use (*P*≤.001) |  |  |  |  |  |
| Hoogenbosch et al [24] | Netherlands | | In ambulances of the University Medical Center Utrecht | 141 | 298 | Users: younger than nonuser (*P*=.02) | NS | NA | NS | Users were less often retired (*P≤*.001) | NA | Patients indicated being users | * * * * |
| Lockwood et al [44] | United States | | Patients before and after kidney transplantation | 64 | 176 | NS | NS | NS | Users: a college education or higher or some college  (*P*=.03) | NA | NA | Patients indicated being users | * * * |
| Martinez et al [45] | Argentina | | Tertiary care hospital | 35,544 | 86,462 | Younger users (*P*≤.001)  After adjusting the age = significant association to PHR^i^ use (*P*≤.001) | User: females  After adjusting the sex remained with the same direction and significant association to PHR use (*P*≤.001) | NA | NA | NA | NA | Entered at least once between September 3 and December 3 of 2012 | * * * |
| McFarland et al [26] | United States | | Radiologic results in the EMR^j^ system in the hospital | 138,841 | 285,999 | Users were older (*P*<.001) | Users were likely to be female (*P*<.001) | Users had a higher annual household income (*P*<.001) | Users had higher levels of educational attainment (*P*<.001) | NA | NA | Enrolled in the portal | * * * * * |
| Neves et al [28] | United Kingdom | | No specific setting | 447 | 205 | NS | NS | NA | Higher educational degree had higher odds of being a portal user (crude OR 1.48, 95% CI 1.00-2.20) (adjusted OR 1.58, 95% CI 1.04-2.39) | NA | NA | Logged in at least once during the study period | * * * |
| Nielsen et al [46] | United States | | Patients with multiple sclerosis | 120 | 120 | Users were younger (*P*≤.001)  Logistic regression: NS | NS | NA | NA | NS | NA | Used the portal’s messaging feature at least once in the years 2008 and 2009 | * * * * |
| Ochoa et al [47] | United States | | Patients who presented to UF^k^ Health | 5648 | 7835 | Logistic regression: users were significantly younger (vs nonusers) | User: females (*P*<.001) | NA | NA | NA | NA | Ever logged in to use the service during the study period | * * * |
| Owolo et al [48] | United States | | Patients with a spine-related condition treated surgically | 6409 | 1546 | Users were younger  Age<65 y (OR 2.00, 95% CI 1.69-2.37, *P*≤.001) | Users were female (3324/6409, 51.9% vs 663/1546, 42.9%)  Female  (OR 1.70, 95% CI 1.49-1.93, *P*≤.001) | NA | NA | Users: employed full time (2001/6409, 31.2% vs 249/1546, 16.1%) and employed (OR 1.39, 95% CI 1.17-1.65, *P*<.001) | Users were married (4531/6409, 70.7% vs 858/1546, 55.5%), in a domestic partnership (OR 1.91, 95% CI 1.68-2.18, *P*≤.001) | Have activated and used PP at the time of their procedure | * * * * * |
| Plate et al [25] | United States | | Patients who underwent primary THA and TKA | 4623 | 1803 | Users: young (*P*<.001) | NS | NA | NA | Users were employed (*P*<.001) | Users were more likely to be married (*P*<.001) | Registration and at least a single login within 1 year | * * * |
| Tome et al [42] | United States | | Adult patients with non–dialysis-dependent CKD^l^ were invited to participate in the study if they visited a nephrology clinic | 159 | 76 | In logistic regression model NS | In logistic regression model NS | Lower income=less likely to use the portal (OR 0.28, 95% CI 0.13-0.60 for those making <US $25,000 annually and OR 0.26, 95% CI 0.12-0.54, for those making US $25,000-US $50,000 annually compared with ≥US $50,000) | Less formal education=less likely to use the portal (OR 0.06, 95% CI 0.01-0.36) | NA | NA | Ask patients whether they use the PP, if yes, then users | * * * * * |
| Ukoha et al [49] | United States | | All women who received prenatal care | 2530 | 920 | Users were younger (*P*<.001)  Multivariable analysis: NS | NA | Low household income=less likely to use (OR 0.91, 95% CI 0.79-1.05)  Multivariable analysis: NS | NA | NA | NA | Active users were those who had sent 1 or more secure messages | * * * * |
| **Wedd et al [29]** | United States | | Patients who received a single-organ kidney or liver transplant at a large transplant center | 252 | 203 |  |  | NA |  |  |  | Whether a patient had any recorded activity at any point during the study period | * * * * * |
|  | Kidney |  |  |  |  | NS | NS |  | Users had college or graduate school education (110/147, 74.8% vs 51/147, 34.7%, *P*<.001).  Users had a college or graduate degree (adjusted risk ratio 1.16, 95% CI 1.01-1.32) | Users were employed (130/179, 72.6%, vs 120/270, 44.4%, *P*<.001) | Users were married (147/243, 60.5% vs 104/208, 50%, *P*=.03) |  |  |
|  | Liver |  |  |  |  | NS | NS |  | Users had college or graduate school education (36/51, 71% vs 40/109, 36.7%, *P*<.001)  Users had college or graduate degree (adjusted risk ratio 1.36, 95% CI 1.01-1.84) | Users were employed (16/22, 73% vs 103/221, 46.6%, *P*=.02) | NS |  |  |

^a^MMAT: Mixed Methods Appraisal Tool (Stars are awarded according to the quality of the study. 5 stars denote the highest rating).

^b^OR: odds ratio.

^c^NA: no data collected.

^d^NS: not statistically significant.

^e^PP: patient portal.

^f^AOR: adjusted odds ratio.

^g^THA: total hip arthroplasty.

^h^TKA: total knee arthroplasty.

^i^PHR: personal health record.

^j^EMR: electronic medical record.

^k^UF: University of Florida.

^l^CKD: chronic kidney disease.
